# Diagnostic accuracy of retrospective application of the Vesical Imaging-Reporting and Data System: preliminary results

**DOI:** 10.1590/0100-3984.2019.0063

**Published:** 2020

**Authors:** André Vaz, Mauricio Zaparolli

**Affiliations:** 1 Hospital Nossa Senhora das Graças, Curitiba, PR, Brazil.; 2 DAPI - Diagnóstico Avançado Por Imagem, Curitiba, PR, Brazil.

**Keywords:** Urinary bladder neoplasms, Data systems, Magnetic resonance imaging/methods, Diffusion magnetic resonance imaging/methods, Muscle, smooth/diagnostic imaging, Neoplasm invasiveness

## Abstract

**Objective:**

To evaluate the retrospective accuracy of the Vesical Imaging-Reporting and Data System (VI-RADS) in detecting muscle invasion in bladder cancer.

**Materials and Methods:**

We investigated patients who underwent pelvic magnetic resonance imaging and were submitted to transurethral resection of a bladder tumor between 2015 and 2018. Thirty cases were reviewed by radiologists blinded to the final clinical stage. The VI-RADS score was applied and compared with the histopathological findings in the surgical specimen.

**Results:**

Of the 30 patients with suspicious bladder lesions, 5 (16.6%) had benign histopathological findings, 17 (56.6%) had non-muscle-invasive bladder cancer, and 8 (26.6%) had muscle-invasive bladder cancer. The optimal criterion to detect muscle-invasive bladder cancer was a final VI-RADS score > 3, for which the sensitivity and specificity were 100% (95% CI: 56.0-100%) and 90.9% (95% CI: 69.3-98.4%), respectively.

**Conclusion:**

The VI-RADS appears to estimate correctly the degree of muscle invasion in suspicious bladder lesions. However, prospective studies evaluating larger samples are needed in order to validate the method.

## INTRODUCTION

Bladder cancer is the most common tumor of the urinary tract, the main types being urothelial and transitional cell carcinoma^(1-3)^. Urothelial carcinoma is divided morphologically into two categories^(2,4-6)^: non-muscle-invasive bladder cancer (NMIBC) and muscle-invasive bladder cancer (MIBC). Whereas NMIBC is limited to the mucosa and lamina propria, typically having a good prognosis, despite the high recurrence rate^(4)^, MIBC has a poor prognosis due to local organ invasion and metastases^(1,7)^.

The appropriate management of urothelial carcinoma depends on accurate detection, staging, and surveillance^(2,3)^. Bladder cancer is staged using the tumor-node-metastasis (TNM) system^(8)^. Detection of muscle invasion (> T1 disease) and metastatic disease are critical to guiding decisions regarding the treatment^(2-4,6,9-11)^. 

Histopathological analysis of the specimen obtained through transurethral resection of bladder tumor (TURBT) can distinguish superficial from invasive tumors, although it is not able to determine the extravesical extent of the tumor^(12)^. Computed tomography of the abdomen and pelvis is the standard imaging method for staging MIBC and detecting extravesical extension (T3 or T4 disease); however, it has low sensitivity for detecting tumors within the bladder and does not allow muscle invasion (T1 or T2 disease) to be quantified^(3)^.

Multiparametric magnetic resonance imaging (mp MRI)-conventional sequences, including T1- and T2-weighted imaging (T2WI), plus two or more functional sequences, such as contrast-enhanced and diffusion-weighted imaging (DWI)-provides better contrast resolution, which can facilitate the distinction between the tumor and adjacent tissues, thereby favoring the identification of muscle invasion^(3,4,6)^. Two recent meta-analyses showed that MRI has high accuracy in predicting muscle invasion^(13,14)^, with a pooled sensitivity of 87% and 92%, respectively, and a pooled specificity of 79% and 88%, respectively. In another meta-analysis^(15)^, specificity was found to be higher when a 3.0 T scanner was used than when a 1.5 T scanner was used-93% versus 83%-and sensitivity and specificity were both found to be higher when mpMRI was used-94% and 95%, respectively. Hence, mpMRI may play a significant role in the treatment decision-making process.

Despite the superior contrast resolution of mpMRI, radiologists may disagree on the presence of muscle invasion^(11)^. Therefore, a method establishing systematic evaluation could standardize imaging reports, increase interobserver agreement, and facilitate communication between specialists. In response to this need, Panebianco et al.^(11)^ proposed the Vesical Imaging-Reporting and Data System (VI-RADS), a standardized mpMRI protocol and scoring system to estimate the risk of muscle invasion by bladder cancer. The VI-RADS score uses T2WI structural categorization, DWI, and dynamic contrast-enhanced MRI to estimate the risk of muscle invasion.

The standardization of terms in imaging reports is essential to ensure adequate therapeutic decision-making, because it allows the medical staff to interpret the imaging findings more accurately and consistently. Therefore, the purpose of this retrospective study was to evaluate the muscle invasion criteria for bladder cancer described in the VI-RADS, correlating its final score with the TURBT histopathological findings. Barchetti et al.^(2)^ and Wang et al.^(16)^ recently demonstrated that the VI-RADS score has high accuracy in differentiating NMIBC from MIBC. To our knowledge, the Barchetti et al.^(2)^ and Wang et al.^(16)^ studies, together with the present study, are the earliest attempts to validate the recently proposed VI-RADS classification of bladder cancer.

## MATERIALS AND METHODS

This was a retrospective study of patients who underwent pelvic mpMRI at a private medical imaging clinic and TURBT at a private referral hospital between August 2015 and August 2018. This study complied with the declaration of Helsinki and Brazilian National Health Council Resolution no. 196/96 regarding research involving humans. Because of the retrospective nature of this study, the need for informed consent was waived.

All mpMRI studies were performed in a 1.5 T scanner (Magnetom Avanto; Siemens Healthineers AG, Munich, Germany) and reviewed with the picture archiving and communication system of the private medical imaging clinic. Because the study was retrospective, the imaging protocol employed was similar but not identical to that suggested in the VI-RADS^(11)^. The main differences between the imaging protocols are summarized in Table 1.

**Table 1:** Differences between the pelvic imaging protocol used in the present study and that recommended in the VI-RADS.

Protocol employed in this study*	VI-RADS protocol(11)
Axial DWI/ADC mapping with b values of 50, 400, and 800 s/mm^2^	Axial and coronal or sagittal DWI/ADC breath-hold spin echo-planar imaging sequence com-bined with spectral fat saturation and a high b value (800-1000 s/mm^2^)
Axial, sagittal, and coronal T2-weighted TSE sequences without fat suppression	T2-weighted TSE or FSE sequences, without fat suppression, in at least two planes (axial, sagittal, or coronal)
Axial T1-weighted sequence with fat suppression	3D SE acquisitions (e.g., SPACE, CUBE, and VISTA) may be included
Contrast-enhanced axial T1-weighted sequence, with fat suppression, acquired at 60 s and at 5 min (in the excretory phase)	T1-weighted GRE (VIBE, LAVA, or THRIVE) sequence, with fat suppression, in 2D or 3D (3D is preferred), acquired before and 30 s after contrast injection, with new acquisitions every 30 s thereafter

* Standard pelvic imaging protocol employed at the private medical imaging clinic. ADC, apparent diffusion coefficient; TSE, turbo spin-echo; FSE, fast spin-echo; 3D, three-dimensional; SE, spin-echo; SPACE, sampling perfection with application-optimized contrasts using different flip angle evolution; VISTA, volume isotropic turbo spin-echo acquisition; CUBE, same as VISTA (by General Electric); 2D, two-dimensional; VIBE, volumetric interpolated breath-hold examination; LAVA, liver acquisition with volume acceleration; THRIVE, T1-weighted high resolution isotropic volume examination; GRE, gradient-recalled echo.

The inclusion criteria were as follows: a suspicious bladder lesion, defined in the VI-RADS as an intravesical lesion with an intermediate signal on T2WI, restricted diffusion on DWI, and early contrast enhancement on dynamic contrast-enhanced MRI^(11)^; and suspicious focal bladder wall thickening (despite the VI-RADS recommendation to include only untreated patients, post-TURBT patients with suspicious focal bladder wall thickening were also included because of a recurrent clinical situation in which mpMRI is requested to assess residual disease after the procedure). To simulate the clinical situation of VI-RADS application, we included cases with and without bladder neoplasia, as identified by TURBT, that met the criteria previously described, given that the use of VI-RADS is intended for pre-TURBT setting^(11)^. Therefore, not all suspicious lesions necessarily correspond to neoplasia. The inclusion of cases without TURBT-confirmed neoplasia differs from the methodology employed in other studies aimed at validating the VI-RADS^(2,16)^. The exclusion criterion was image quality being insufficient to allow the determination of the VI-RADS score.

All TURBT procedures were performed by the urology department at the referral hospital. The TURBT histopathological findings were evaluated by one of the pathologists at that hospital, with more than 14 years of experience and a special interest in abdominal pathology. The histopathology slides were not reviewed. The MRI scans were evaluated separately by a radiology resident and by an expert in abdominal radiology from the imaging clinic with 13 years of experience and a special interest in urogenital oncology imaging, both of whom were blinded to the final clinical stage and worked independently. In all cases, each researcher applied the VI-RADS muscle invasion criteria^(11)^. When patients presented multiple bladder lesions, the dominant lesion (that with the highest VI-RADS score) was considered. All images were then jointly reviewed by both researchers. Discrepancies regarding the final VI-RADS score were resolved by consensus, and the final score was compared with the histopathological results. Details of the scoring system can be found in the original description^(11)^.

In the statistical analysis, Microsoft Excel was used in order to calculate the means, standard deviations, proportions, sensitivities, specificities, positive predictive values, negative predictive values, and the empirical receiver operating characteristic (ROC) curve. The Newcombe-Wilson method was used in order to calculate 95% confidence intervals (CIs), and Fisher’s exact test was used in order to assess statistical significance.

## RESULTS

Thirty-eight pelvic mpMRI scans met the inclusion criteria. However, eight cases were excluded because of insufficient image quality, mostly due to inadequate bladder distention (in 75%). Two (25%) of those eight cases were eventually diagnosed with MIBC, despite having no suspicious mpMRI findings other than diffuse bladder wall thickening (which was believed to be inflammatory in nature). Therefore, the final sample comprised 30 pelvic mpMRI scans (of 30 patients), all of which were reviewed with the purpose of applying the VI-RADS muscle invasion criteria and comparing the VI-RADS score with the TURBT histopathological results. Of the 30 patients, 19 (63.3%) were male and 11 (36.6%) were female. The mean age was 68 ± 8.8 years.

The pathology findings in the TURBT surgical specimen included the following: vascular congestion and edema of the lamina propria, in 1 (3.3%) of the cases; prostatic tissue fragment, in 1 (3.3%); atypical urothelial proliferation, in 3 (10%); low grade papillary urothelial carcinoma, in 3 (10%); noninvasive high-grade papillary urothelial carcinoma, in 9 (30%); high-grade papillary urothelial carcinoma with subepithelial invasion but without muscle invasion, in 5 (16.6%); and muscle-invasive urothelial carcinoma, in 8 (26.6%).

The mean time from TURBT to image acquisition was 29 ± 19 days (range, 5-67 days). The site most often affected was the anterior bladder wall (in 30%), followed by the posterior wall (in 18.5%) and trigone (in 14.8%). The mean size of the suspicious lesions was 21.3 ± 19.6 mm (range, 3-86 mm).

**Figure 1:**
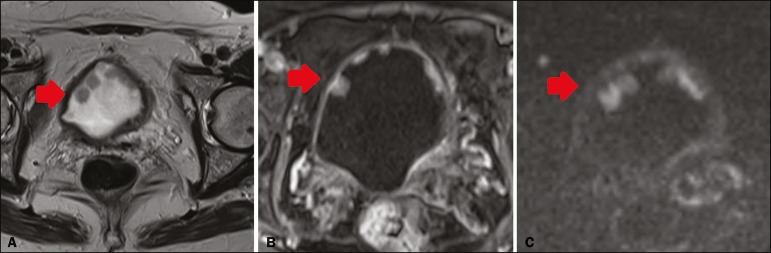
A 73-year-old woman with multiple polypoid VI-RADS 2 lesions in the anterior bladder wall. The dominant lesion (arrow) presents a high-signal-intensity thickened inner layer and integrity of the adjacent muscularis propria on T2WI (**A**), early enhancement of the inner layer on dynamic contrast-enhanced MRI (**B**) and a low-signal-intensity stalk on DWI (**C**). Histopathological analysis of the lesion indicated high-grade papillary urothelial carcinoma with an area of invasion of subepithelial tissue and normal adjacent muscularis propria bundles.

**Figure 2:**
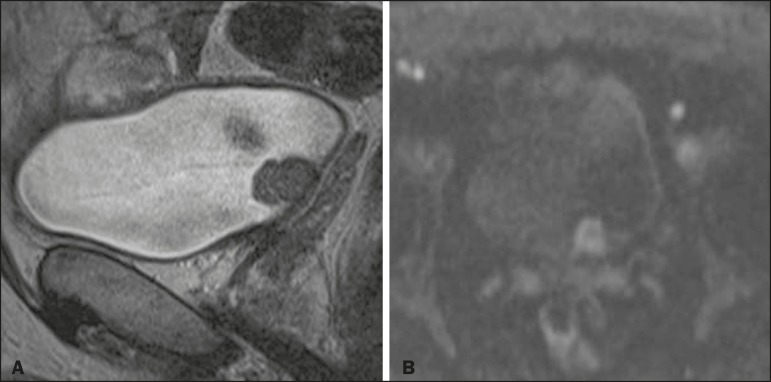
A 66-year-old man with a polypoid VI-RADS 3 lesion in the posterior bladder wall, without a high-signalintensity thickened inner layer but with no clear disruption the muscularis propria on T2WI (**A**), a high-signal-intensity inner layer in DWI (**B**), and no clear disruption of the muscularis propria in the 5-min excretory phase (not shown, early contrast phase not recorded). Histopathological analysis of the lesion indicated high-grade papillary urothelial carcinoma with foci of invasion of the subepithelial tissue and normal adjacent muscularis propria bundles.

**Figure 3:**
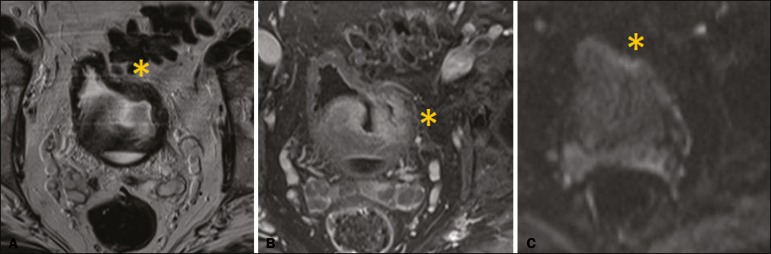
A 71-year-old man with post-TURBT focal thickening of the left lateral wall of the bladder, presenting interruption of the muscularis propria low-signal-intensity line, suggesting muscle infiltration on T2WI (asterisk in **A**), focal enhancement extending into the muscularis propria in dynamic contrast-enhanced MRI (asterisk in **B**) and a tiny focus of restricted diffusion in the muscularis propria (asterisk in **C**). The minimal bladder distention significantly impeded the evaluation of the lesion. Although most of the intravesical vegetative lesion was removed during TURBT, signs of remaining suspicious lesion persist with signs of extension to the muscularis propria, without obvious extravesical extension. We suggest that such lesions could be classified as VI-RADS 4 (muscle invasion likely). Histopathological analysis of the lesion indicated high-grade urothelial carcinoma with subepithelial and muscle invasion.

**Figure 4:**
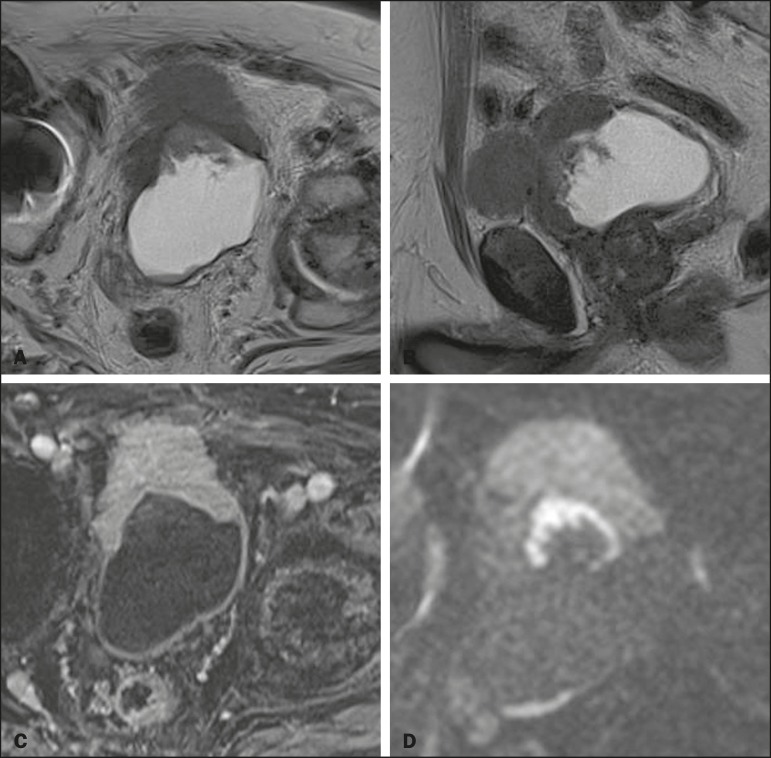
An 86-year-old man with a post-TURBT VI-RADS 5 infiltrative lesion in the anterior bladder wall, presenting evident extravesical extension on T2WI (**A,B**), early enhancement extending to the extravesical fat (**C**), and high signal intensity, also extending to the extravesical fat, on DWI (**D**). Although most of the intravesical vegetative lesion was removed during TURBT, an extensive lesion persisted, allowing the characterization of MIBC. Histopathological analysis of the lesion indicated invasive high-grade papillary urothelial carcinoma with invasion of subepithelial tissues and infiltration of muscularis propria.

In 8 (26.7%) of the 30 cases, the final VI-RADS score assigned was 1-indicating that muscle invasion is highly unlikely-, whereas it was 2-indicating that muscle invasion is unlikely (Figure 1)-in 10 (33.3%), 3-indicating that muscle invasion is questionable (Figure 2)-in 3 (10.0%), 4-indicating that muscle invasion is likely (Figure 3)-in 2 (6.7%), and 5-indicating that muscle invasion and invasion of other extravesical tissues is highly likely (Figure 4)-in 7 (23.3%). In relation to morphology, 13 (43.3%) of the lesions were sessile, 9 (30%) were polypoid, and 8 (26.7%) presented as a focal wall thickening, the last not being included in the original VI-RADS structural description and related to the fact that the patients had previously undergone bladder biopsy or TURBT.

**Figure 5:**
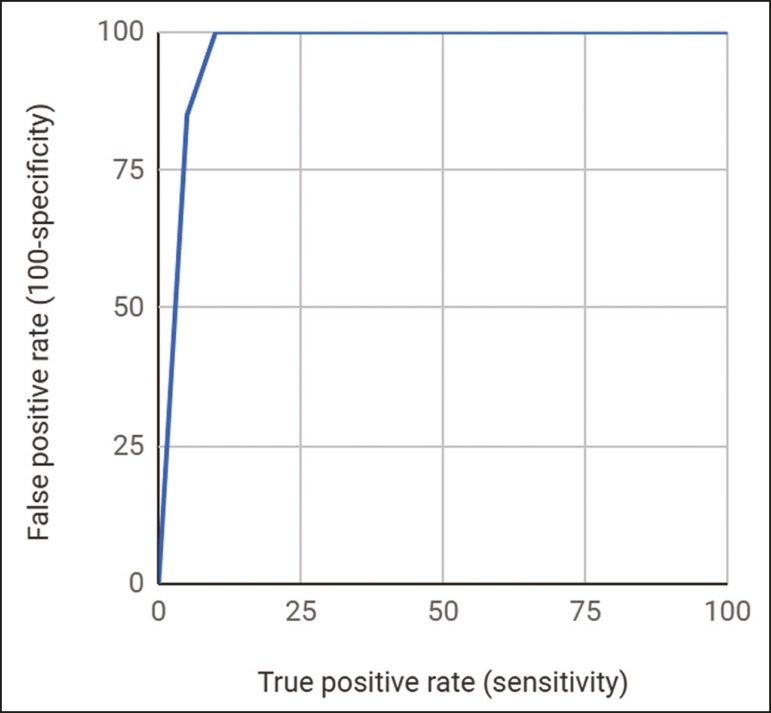
Empirical ROC curve.

All of the lesions receiving a final VI-RADS score of 1, 2, or 3 were free of muscle invasion, whereas muscle invasion was identified in 50% of those receiving a final VI-RADS score of 4 and in 85.7% of those receiving a final VI-RADS score of 5. The optimal criterion to detect MIBC using Youden’s index was a final VI-RADS score > 3, which had a sensitivity and specificity of 100% (95% CI: 56.0-100%) and 90.9% (95% CI: 69.3-98.4%), respectively (*p* < 0.05). Details, including the positive predictive value and negative predictive value for each cutoff score, are summarized in Table 2. The area under the ROC curve was 0.9675, and the ROC curve analysis is summarized in Figure 5. Misdiagnosis occurred in two cases (6.7%), both of which were cases of overstaging. In both cases, the time from TURBT to image acquisition was > 2 weeks (32 days and 38 days, respectively).

**Table 2:** Sensitivity, specificity, positive predictive value, and negative predictive value for each final VI-RADS score cutoff point.

	Sensitivity			Specificity			Positive predictive value			Negative predictive value		
Final VI-RADS score	Value 95% CI			Value 95% CI			Value 95% CI			Value 95% CI		Accuracy
> 2	100%	56.0-100%		36.3%	18.0-59.1%		33.3%	15.4-56.8%		100%	59.7-100%	51.7%
> 3	100%	56.0-100%		77.2%	54.1-91.3%		58.3%	25.5-83.5%		100%	77.0-100%	82.7%
> 4	100%	56.0-100%		90.9%	69.3-98.4%		77.7%	40.1-96.0%		100%	79.9-100%	93.1%
5	85.7%	42.0-99.2%		95.4%	75.1-99.7%		85.7%	42.0-99.2%		95.5%	75.1-99.7%	93.1%

## DISCUSSION

Recent studies conducted in Brazil have highlighted the importance of imaging methods, particularly MRI, in the evaluation of tumors affecting the genitourinary system^(17-22)^. The reported accuracy of mpMRI for tumor staging ranges from 75% to 94.5%^(9,10,12,15)^. Although interobserver agreement is usually good^(12)^, radiologists may disagree on muscle invasion in some cases^(11)^, especially when there is minimal bladder wall thickening, concomitant inflammatory changes^(9)^, or insufficient contrast between the tumor and the bladder wall in terms of signal intensity^(10)^. Therefore, the VI-RADS has been promoted as a means of ensuring adequate interobserver agreement in the estimation of muscle invasion^(11)^.

Our preliminary results in this small sample of suspicious bladder lesions indicate that the performance of the VI-RADS is satisfactory, given that sensitivity, specificity, and overall accuracy were high. However, to validate the method, there is a need for further studies, involving larger samples, preferably prospective, multicenter studies, with estimation of inter-reader agreement. Two recent retrospective studies evaluated the accuracy of the VI-RADS in distinguishing NMIBC from MIBC^(2,16)^. In a study including 75 patients and employing a 3.0 T scanner, Barchetti et al.^(2)^ demonstrated that the VI-RADS has high accuracy, with a sensitivity of 82-91% and a specificity of 85-89% (two readers), although the authors applied a final VI-RADS score cutoff of > 2. In that study, the overall level of interobserver agreement for the final VI-RADS score was good. In another study, including 75 patients and also employing a 3.0 T scanner, Wang et al.^(16)^ reported that disagreements between the readers were resolved by consensus, as in our study. Those authors also found that a final VI-RADS score cutoff of > 2 had high accuracy-with a sensitivity of 87.1% and a specificity of 96.5%-and that the overall level of interobserver agreement was excellent.

As reported by other authors, the most common type of misdiagnosis in the present study was overstaging. However, overstaging occurred in a significantly lower proportion of the patients in our sample (6.7%), compared with the 34% and 32% reported by Kim et al.^(9)^ and Tekes et al.^(12)^, respectively, probably due to the good contrast resolution of DWI^(10)^.

The original structural description in the VI-RADS includes only exophytic (polypoid) and sessile (broad-based) tumors^(11)^. However, a significant proportion (26.6%) of the patients in our sample underwent MRI only after bladder biopsy or TURBT, and the MRI scan showed suspicious focal bladder wall thickening in all of those patients. Therefore, we suggest that suspicious focal bladder wall thickening be included in the VI-RADS categorization of morphology. In such cases, to avoid overestimation of extravesical extension due to post-biopsy inflammatory changes, we recommend an interval of 1-2 weeks between the bladder procedure and MRI acquisition^(1,11)^.

Despite previous reports stating that the degree of bladder distention has no influence on the detection of bladder tumors^(9)^, the major difficulty in applying the VI-RADS score was evaluating bladder lesions in a minimally distended bladder (Figure 4). The use of the protocol devised by Panebianco et al.^(11)^ should resolve that issue.

The preliminary results related to the VI-RADS reporting criteria for muscle invasion in bladder cancer obtained in the present study are promising. However, full application of the method may require some modification of MRI pelvic imaging protocols. Therefore, to evaluate the method further, we encourage the adoption of the standardized mpMRI protocol devised by Panebianco et al.^(11)^.

## CONCLUSION

The VI-RADS grading system appears to estimate correctly the degree of muscle invasion in suspicious bladder lesions and could provide a means of standardizing radiology reports and reducing interobserver variability. In addition to the original morphological VI-RADS descriptions, we suggest inclusion of suspicious focal bladder wall thickening to describe lesions in patients who have previously undergone biopsy or TURBT.

## References

[1] Wong-You-Cheong JJ, Woodward PJ, Manning MA (2006). Neoplasms of the urinary bladder: radiologic-pathologic correlation. Radiographics.

[2] Barchetti G, Simone G, Ceravolo I (2019). Multiparametric MRI of the bladder: inter-observer agreement and accuracy with the Vesical Imaging-Reporting and Data System (VI-RADS) at a single reference center. Eur Radiol.

[3] Rose TL, Lotan Y (2018). Advancements in optical techniques and imaging in the diagnosis and management of bladder cancer. Urol Oncol.

[4] Verma S, Rajesh A, Prasad SR (2012). Urinary bladder cancer: role of MR imaging. Radiographics.

[5] Babjuk M, Böhle A, Burger M (2017). EAU guidelines on non-muscle-invasive urothelial carcinoma of the bladder: update 2016. Eur Urol.

[6] Alfred Witjes J, Lebret T, Compérat EM (2017). Updated 2016 EAU guidelines on muscle-invasive and metastatic bladder cancer. Eur Urol.

[7] Lee CH, Tan CH, Fari SC (2017). Role of imaging in the local staging of urothelial carcinoma of the bladder. AJR Am J Roentgenol.

[8] American Joint Committee on Cancer (2017). AJCC cancer staging manual.

[9] Kim B, Semelka RC, Ascher SM (1994). Bladder tumor staging: comparison of contrast-enhanced CT, TI- and T2-weighted MR imaging, dynamic gadolinium-enhanced imaging, and late gadolinium-enhanced imaging. Radiology.

[10] Takeuchi M, Sasaki S, Ito M (2009). Urinary bladder cancer: diffusion weighted MR imaging-accuracy for diagnosing T stage and estimating histologic grade. Radiology.

[11] Panebianco V, Narumi Y, Altun E (2018). Multiparametric magnetic resonance imaging for bladder cancer: development of VI-RADS (Vesical Imaging-Reporting And Data System). Eur Urol.

[12] Tekes A, Kamel I, Imam K (2005). Dynamic MRI of bladder cancer: evaluation of staging accuracy. AJR Am J Roentgenol.

[13] Gandhi N, Krishna S, Booth CM (2018). Diagnostic accuracy of magnetic resonance imaging for tumour staging of bladder cancer: systematic review and meta-analysis. BJU Int.

[14] Woo S, Suh CH, Kim SY (2017). Diagnostic performance of MRI for prediction of muscle-invasiveness of bladder cancer: a systematic review and meta-analysis. Eur J Radiol.

[15] Huang L, Kong Q, Liu Z (2018). The diagnostic value of MR imaging in differentiating T staging of bladder cancer: a meta-analysis. Radiology.

[16] Wang H, Luo C, Zhang F (2019). Multiparametric MRI for bladder cancer: validation of VI-RADS for the detection of detrusor muscle invasion. Radiology.

[17] Leão LRS, Mussi TC, Yamauchi FI (2019). Common pitfalls in renal mass evaluation: a practical guide. Radiol Bras.

[18] Baghdanian AA, Kim YJ, Baghdanian AH (2019). Differences in negative predictive value of prostate MRI based in men with suspected or known cancer. Radiol Bras.

[19] Tibana TK, Santos RFT, Said LAM (2019). Fibroepithelial polyp of the ureter: the value of magnetic resonance imaging of the urinary tract in diagnosis and therapeutic planning. Radiol Bras.

[20] Baroni RH (2019). Can biopsy be avoided in patients with clinical suspicion of prostate cancer and a negative result on multiparametric magnetic resonance imaging?. Radiol Bras.

[21] Tames AVC, Fonseca EKUN, Yamauchi FI (2019). Progression rate in Bosniak category IIF complex renal cysts. Radiol Bras.

[22] Miranda CLVM, Sousa CSM, Bastos BB (2018). Giant renal angiomyolipomas in a patient with tuberous sclerosis. Radiol Bras.

